# HCF-YOLO: High-Resolution Center-Guided Feature Fusion for Dense Cotton Seedling Detection in UAV Imagery

**DOI:** 10.3390/s26216842

**Published:** 2026-10-29

**Authors:** Yantao Li, Hongbo Su, Wenbin Zhang, Yunjie Zhao, Yuxiang Wang

**Affiliations:** 1 College of Computer and Information Engineering, Xinjiang Agricultural University, Urumqi 830052, China; 2 Xinjiang Jinfengyuan Seed Industry Co., Ltd., Wensu County, Aksu Prefecture 843100, China; 3 Engineering Research Center for Intelligent Agriculture, Ministry of Education, Urumqi 830052, China; 4 Xinjiang Agricultural Informatization Engineering Technology Research Center, Xinjiang Agricultural University, Urumqi 830052, China; 5 College of Agriculture, Xinjiang Agricultural University, Urumqi 830052, China

**Keywords:** UAV imagery, cotton seedling detection, small-object detection, feature fusion, center guidance, model compression

## Abstract

Accurate estimates of cotton seedling abundance and spatial distribution support emergence assessment and field management. However, seedlings occupy few pixels in unmanned aerial vehicle (UAV) imagery, occur at high densities, and frequently overlap. This study proposes a High-Resolution Center-Guided Feature Fusion model (HCF-YOLO) that combines a P2 detection layer, Lightweight Multi-Scale Feature Fusion (LMSFF), and training-only Gaussian Center Guidance (GCG). The dataset contained 1040 images and 101,824 annotated cotton seedlings and was divided into 832 training, 104 validation, and 104 test images. On the main test split, HCF-YOLO11n achieved an mAP50 of 0.9354 and an mAP50-95 of 0.4431, improvements of 5.10 and 3.74 percentage points over YOLO11n, while reducing the parameter count and FLOPs by 14.18% and 14.63%. The performance ranking was consistent across three random seeds and was retained in the external-source evaluation. Under the pruning protocol, the 5%-pruned HCF-YOLO11n achieved an mAP50-95 of 0.4578 on the same 104-image test split while reducing parameters and FLOPs by 11.19% and 6.87%, respectively. TensorRT FP16 reduced network inference latency to 1.453 ms, and Raspberry Pi 5 deployment reached 6.67 FPS. Overall, HCF-YOLO balances accuracy, model size, and edge efficiency for UAV-assisted cotton seedling recognition and image-plane localization, although onboard validation remains necessary.

## 1. Introduction

Cotton is a globally important fiber and cash crop that supports textile production, rural livelihoods, and agricultural economies [1]. Its early stand establishment affects subsequent field management, while seedling abundance and spatial distribution provide direct evidence of emergence performance [2]. Conventional surveys rely on manual sampling and ground observations and are therefore labor-intensive. Unmanned aerial vehicle (UAV) imagery offers high spatial resolution, rapid acquisition, and broad coverage [3], making it an important data source for precision agricultural monitoring [4] and cotton seedling surveys [5].

Deep learning has expanded the use of UAV imagery for agricultural object detection, crop counting, and seedling monitoring [6,7]. Plant populations can also be estimated from very-high-resolution aerial imagery by combining vegetation indices, segmentation, and convolutional neural networks [8]. These approaches have produced reliable counts across different crops and seedling stages [9].

High-resolution imagery has been used to estimate plant populations and planting uniformity [10]. UAV red-green-blue (RGB) imagery combined with deep learning has also supported large-scale plant recognition and counting [11]. For cotton seedlings, cross-scale feature enhancement has improved detection under complex field conditions [12], and high-resolution UAV imagery has enabled individual-plant recognition [13]. Regression and region-based counting methods usually provide aggregate counts without localizing individual seedlings. Object detection instead provides instance locations in addition to image-level counts.

Object detection addresses this limitation by returning both plant counts and individual locations. Lu et al. [14] combined YOLOv5 with assisted annotation and rotation augmentation to detect maize across leaf stages. Feng et al. [15] proposed a lightweight YOLOv8n model for small maize seedlings through multi-scale feature extraction. Xu et al. [16] combined Mask R-CNN and YOLOv5 to reduce counting errors caused by overlapping maize leaves. Ren et al. [17] linked deep-learning detections with Voronoi analysis to characterize maize emergence, planting uniformity, interplant distance, and seedling quality.

YOLO-family detectors have also been applied to weeds in cotton fields [18] and small objects in UAV imagery [19]. Lightweight variants extend these applications to resource-constrained UAV platforms [20]. Nevertheless, cotton seedling scenes remain challenging because the targets are small, numerous, and surrounded by complex backgrounds [12]. Similar scale-related difficulties have been reported for cotton boll detection [21]. More generally, dense UAV targets are susceptible to feature attenuation and unstable localization [22]. Two critical limitations arise in dense cotton seedling imagery. First, repeated backbone downsampling can suppress the edges, textures, and spatial cues of seedlings before these features reach deeper layers. Second, responses from adjacent seedlings can overlap, obscuring object centers and shifting predicted boxes. Addressing both limitations requires high-resolution representations and stronger center localization without excessive model complexity.

To address these limitations, we propose the High-Resolution Center-Guided Feature Fusion model (HCF-YOLO) based on YOLO11n. A P2 detection layer preserves shallow texture, edge, and spatial information for small seedlings. Lightweight Multi-Scale Feature Fusion (LMSFF) combines shallow detail with deeper semantic features while limiting redundant computation. Gaussian Center Guidance (GCG) supplies center-region supervision during training to support localization in dense scenes. Evaluation included representative-model comparisons, three-seed repeat training, qualitative and localization analyses, ablation studies, structured pruning, quantized inference, external-source validation, and controlled Raspberry Pi 5 benchmarking.

## 2. Materials and Methods

### 2.1. Cotton Seedling Detection Dataset

The cotton seedling detection dataset combined two public sources: the Cotton Analysis Dataset (COT-AD) [23] and Cotton-BNYQS [24]. After quality control and duplicate screening, the retained COT-AD subset contained 940 UAV images and 63,674 cotton-seedling annotations. Cotton-BNYQS contributed 100 images and 38,150 annotations. The two sources therefore represented 90.38% and 9.62% of the images and 62.53% and 37.47% of the annotations, respectively. The final dataset contained 1040 original images and 101,824 annotated instances. All statistics refer to original images before online data augmentation. Table 1 summarizes the composition of the two data sources.

The metadata distributed with the two public sources did not provide consistent cultivar identifiers. Cotton variety was therefore not treated as a stratification factor or covariate in model training or evaluation. The reported results characterize aggregate performance across the available source imagery, while cultivar-specific robustness remains to be verified.

The 1040 original images were divided into training, validation, and test sets at an 8:1:1 ratio, yielding 832, 104, and 104 images, respectively. Random flipping, scaling, color perturbation, and cropping were applied online only after the training split had been defined. The validation and test images were neither augmented nor expanded. The validation set supported training monitoring and checkpoint selection, whereas the test set was reserved for final evaluation.

The final COT-AD subset comprised 657 images at 8000 × 6000 pixels, 199 images at 4000 × 3000 pixels, and 84 images at 5472 × 3648 pixels. All 100 Cotton-BNYQS images were 640 × 640 pixels. Ground-sampling-distance metadata were unavailable in the redistributed subset. For a consistent object-size analysis, bounding boxes were mapped to the 640 × 640 model-input scale by aspect-ratio-preserving resizing. The median box width and height were 22.23 and 23.19 pixels, with interquartile ranges of 8.00–36.78 and 8.00–39.06 pixels, respectively. Overall, 66.09% of valid boxes had areas below 32 × 32 pixels, confirming that small targets predominated. Three zero-width or zero-height annotation records were excluded only from this descriptive analysis. SHA-256 comparison found no byte-identical images. A conservative 64-bit difference-hash screen used a Hamming-distance threshold of 3 and flagged two candidate pairs. Manual inspection identified one adjacent COT-AD frame pair; one image containing 94 annotations was removed before the final counts and dataset split were produced. The remaining Cotton-BNYQS pair was visually similar but nonidentical.

### 2.2. HCF-YOLO Cotton Seedling Detection Model

#### 2.2.1. Overall Architecture

**Figure 1:**
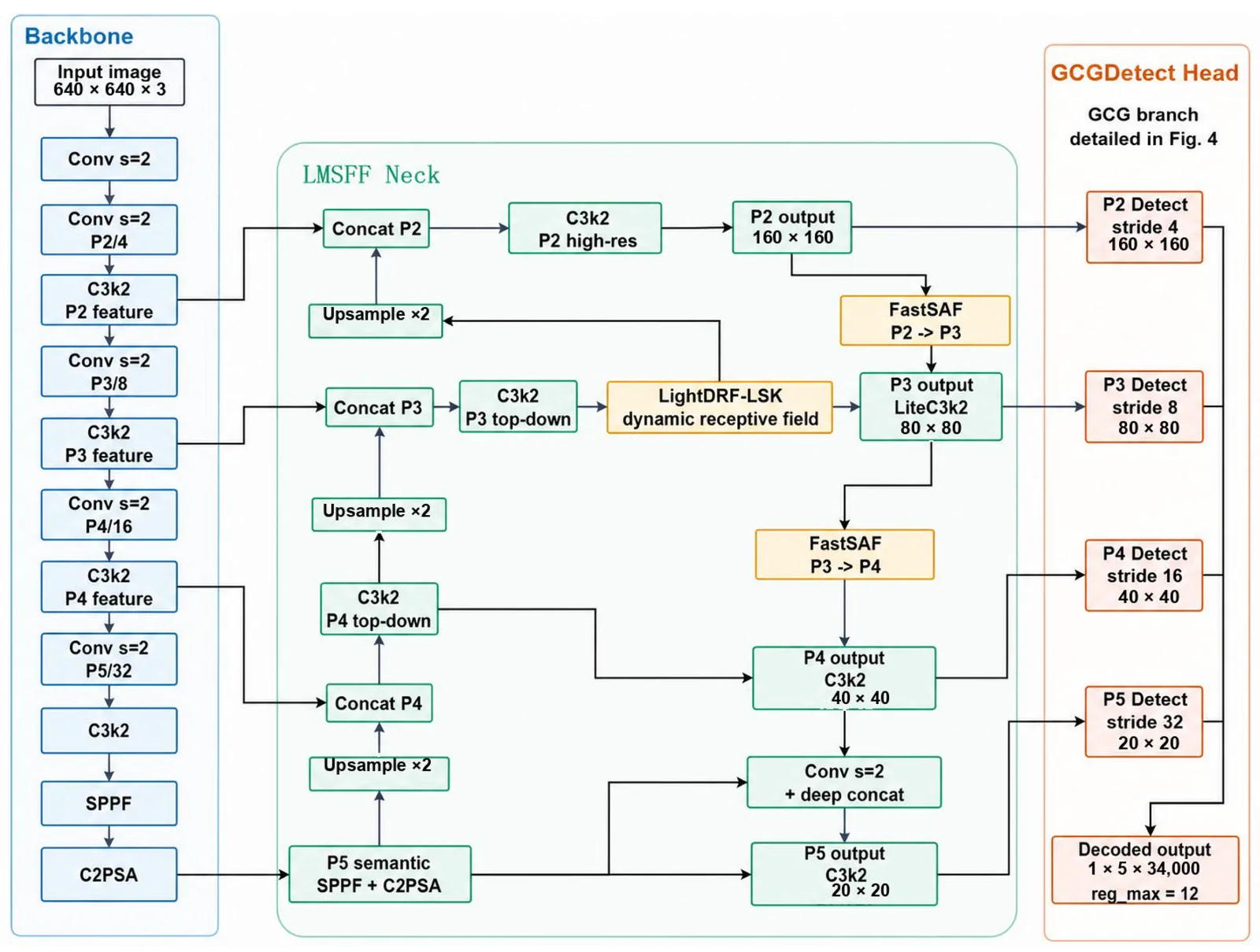
Overall architecture of HCF-YOLO for cotton seedling detection. Abbreviations: HCF-YOLO, High-Resolution Center-Guided Feature Fusion; LMSFF, Lightweight Multi-Scale Feature Fusion; GCG, Gaussian Center Guidance. (Blue: backbone; green: LMSFF; orange: FastSAF; red: GCG/head).

YOLO11n offers a compact architecture and efficient inference for UAV small-object detection [25,26] and was therefore selected as the baseline. Figure 1 presents HCF-YOLO11n, which adds a P2 detection layer, LMSFF, and a center-guidance branch to the YOLO11n backbone. P2 preserves shallow spatial detail, LMSFF integrates information across scales, and GCG supervises seedling centers during training. Because GCG is used only for training, it is absent from the inference graph.

#### 2.2.2. P2 High-Resolution Detection Layer

Cotton seedlings occupy relatively few pixels in UAV images. With each backbone downsampling operation, their effective response area becomes smaller in deeper feature maps. Important edge, texture, and spatial cues may consequently disappear, increasing the likelihood of missed detections and localization offsets. The relation between target size and the number of downsampling operations can be approximated as follows:

(1) $$S_{n}=\frac{S_{0}}{2^{n}},$$

Here, *S*_0_ is the target size in the original image, *S*_n_ is the target size after *n* downsampling operations, and *n* is the number of downsampling operations. The same integer *n* is used throughout Equation (1); no separate *n*-prime term is defined. This relationship shows that repeated downsampling reduces the effective response area of a small seedling in the feature map. Shallow, high-resolution features are therefore important for small-object cotton seedling detection.

**Figure 2:**
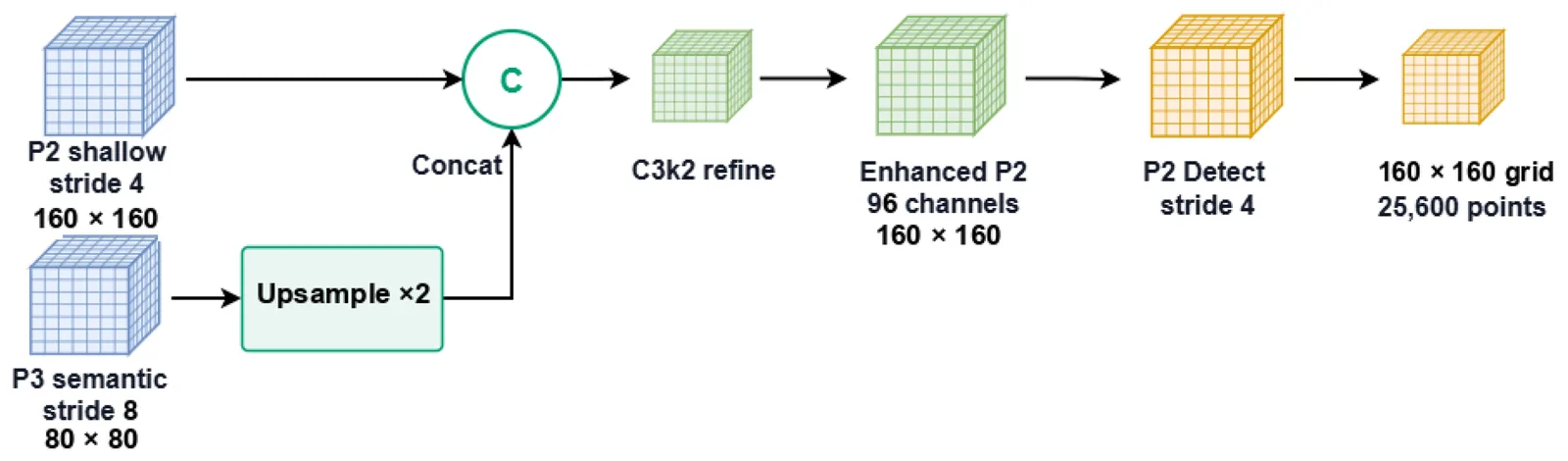
Architecture of the P2 high-resolution detection layer. (Blue: input; green: fusion; orange: output).

Spatial resolution strongly affects early-stage plant detection in UAV imagery [27]. As shown in Figure 2, a P2 high-resolution detection layer was added to the original P3, P4, and P5 layers of YOLO11n. For a 640 × 640 input image, P2 uses a stride of 4 and produces a 160 × 160 feature map. Prediction at this resolution preserves shallow texture, edge, and spatial information, strengthening the representation of small and densely distributed cotton seedlings.

#### 2.2.3. Lightweight Multi-Scale Feature Fusion

**Figure 3:**
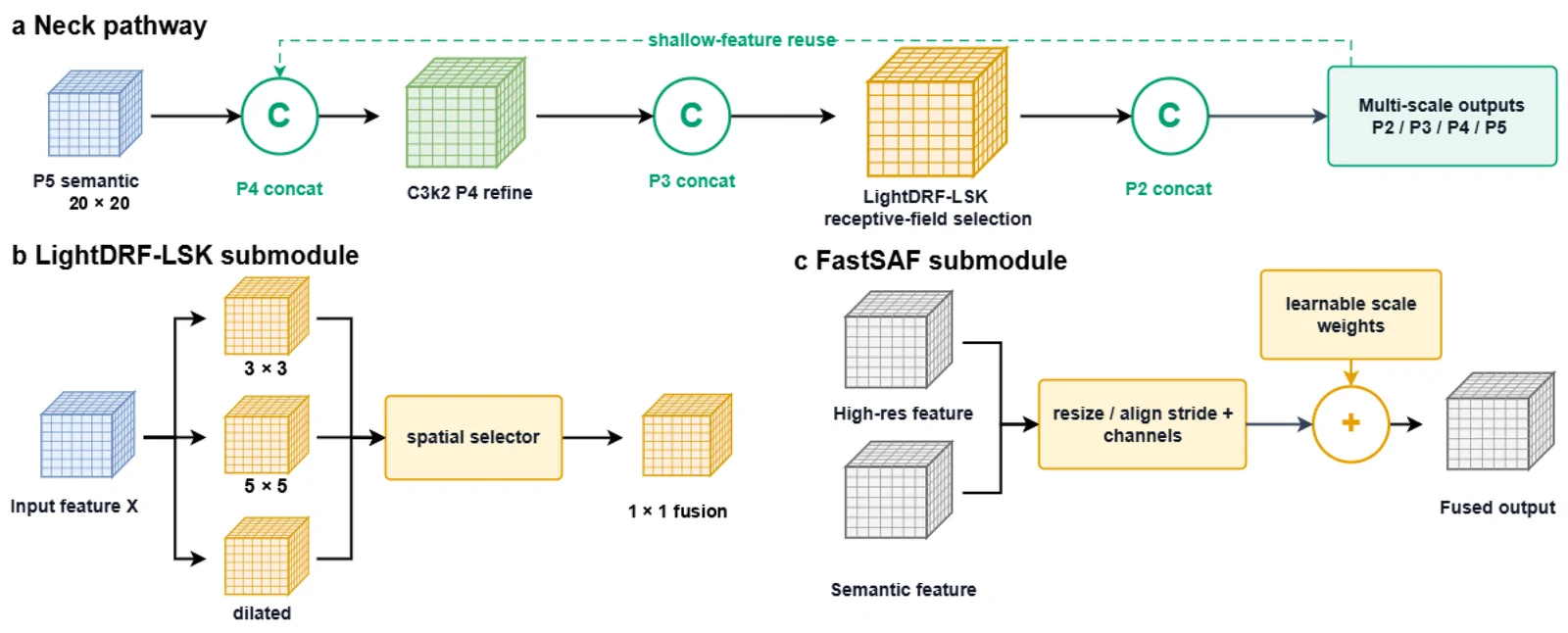
Architectures of the modules used in LMSFF: (**a**) the multi-scale neck pathway; (**b**) the LightDRF-LSK submodule; and (**c**) the FastSAF submodule. (Blue: input; green: neck/output; orange: fusion; gray: aligned/fused; ×: dimensions).

Shallow feature maps retain fine spatial, edge, and texture information, whereas deeper maps provide semantic information for category discrimination. Neither scale alone is sufficient for dense and small cotton seedlings. Figure 3 shows the LMSFF architecture, which combines both forms of information. LMSFF retains high-resolution P2 features and reconstructs the intermediate- and high-level fusion paths with a lightweight configuration to reduce redundant computation.

In the implemented configuration, the top-down path propagates P5 features through P4 and P3 to a stride-4 P2 feature map. Before application of the YOLO11n width multiplier, the nominal output channels of the top-down P4, P3, and P2 blocks are 512, 256, and 96. LightDRF-LSK is placed at P3 and begins with a 1 × 1 bottleneck using an expansion ratio of 0.25. It then applies three depthwise branches: a 3 × 3 kernel with dilation 1, a 3 × 3 kernel with dilation 2, and a 5 × 5 kernel with dilation 2. A 1 × 1 spatial selector generates three softmax-normalized weights. Their weighted sum is expanded through a 1 × 1 convolution and added through a learnable residual scale.

*F_r_* = Conv*_d_*_=*r*_(*F*), (2)

(3) $$F_{\mathrm{DRF}}=\sum_{r\in R}\alpha_{r}F_{r},$$

Here, Conv*_d_*_=*r*_ denotes a convolution with dilation rate *r*. The term *F_r_* is the output of the corresponding receptive-field branch, *α_r_* is its weight, and *R* is the set of branches. Dynamic branch selection preserves fine small-object responses while improving discrimination within dense target regions.

The bottom-up path retains four detection levels, P2–P5, with strides of 4, 8, 16, and 32. The P2 feature is aligned with P3 by a 3 × 3 stride-2 convolution, and the P3-to-P4 transitions use the same alignment operation. FastSAF fuses two adjacent inputs. Nearest-neighbor interpolation aligns the spatial size to the second input, while identity mapping or a 1 × 1 convolution aligns the channels. Two learned scalar weights are softmax-normalized before summation. The nominal fusion outputs are 256 channels at P3 and 512 channels at P4. The final detection head receives nominal P2–P5 channel widths of 96, 256, 512, and 1024.

(4) $$F_{\mathrm{SAF}}=\sum_{i=1}^{N}\omega_{i}F_{i},$$

(5) $$\omega_{i}=\frac{\exp\left(a_{i}\right)}{\sum_{j=1}^{N}\exp\left(a_{j}\right)},$$

Here, *F_i_* is the input feature at scale *i*, and *ω_i_* is the corresponding fusion weight. The term *a_i_* is the learned weight response, and *N* is the number of feature scales involved in fusion. This formulation adaptively balances shallow detail and deep semantic features. It also reduces information redundancy and small-object feature attenuation caused by fixed fusion schemes.

Adding the P2 detection layer preserves high-resolution spatial information but requires processing a 160 × 160 feature map. In the ablation study, the P2-only configuration increased FLOPs and batch-16 network latency by 61.42% and 16.41%, respectively, relative to YOLO11n (Table 4). LMSFF mitigates this overhead through lightweight intermediate channels, depthwise operations, channel alignment, and weighted adjacent-scale fusion. When combined with P2, LMSFF reduced parameters, FLOPs, and latency by 16.66%, 47.11%, and 8.68%, respectively, relative to the P2-only configuration while retaining the high-resolution pathway.

#### 2.2.4. GCG Center-Guidance Strategy

**Figure 4:**
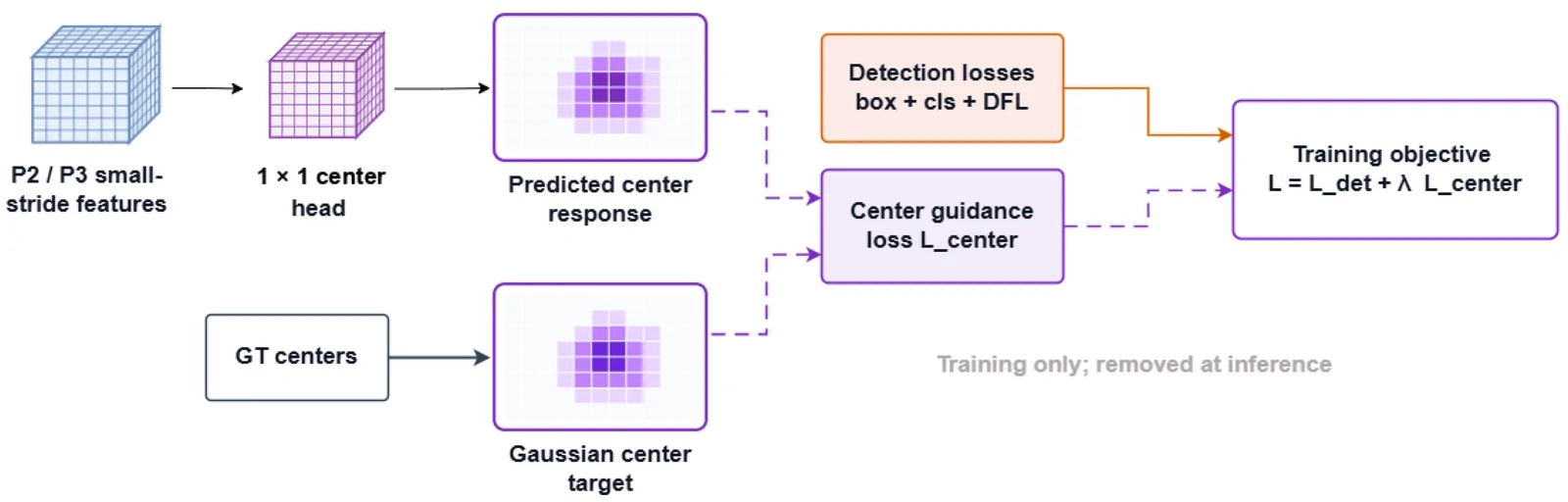
Architecture of the GCG center-guidance strategy. (Blue: features; purple: center guidance; orange: detection loss; dashed purple: training-only path).

Seedling centers are often closely spaced in UAV images of dense cotton stands. Figure 4 shows the training-only GCG branch. Each ground-truth box is mapped to the supervised feature grid using its normalized center and dimensions. The Gaussian radius is calculated as one-half of the smaller box dimension on the feature map, with a lower bound of one pixel. The Gaussian standard deviation is one-third of this radius. Overlapping target Gaussians are combined by an element-wise maximum. Because every Gaussian peaks at one, no additional target-map normalization is applied.

For a ground-truth box center (*c_x_*,*c_y_*), the corresponding Gaussian center-supervision map is defined as follows:

(6) $$G\left(x,y\right)=\exp\left(-\frac{{\left(x-c_{x}\right)}^{2}+{\left(y-c_{y}\right)}^{2}}{2\sigma^{2}}\right),$$

where the coordinates are measured on the corresponding feature map and *σ* is determined from target size as defined above. By default, GCG supervises the two detection levels with the smallest strides. For the four-level HCF-YOLO11n head, these are P2 and P3 at strides 4 and 8. Consecutive 1 × 1 convolutions transform each selected feature into one center-logit map. The center-guidance loss is the mean binary cross-entropy with logits across all pixels and supervised levels. Equation (7) combines the detection and center-guidance losses with a weighting coefficient of 0.1. The box, classification, and distribution-focal-loss gains are 7.5, 0.5, and 1.5, respectively. GCG is disabled during validation, inference, and export, so it introduces no inference-time branch.

*L* = *L_det_* + *λ_g_L_center_* (7)

where *L_det_* is the original detection loss, *L_center_* is the center-guidance loss, and *λ_g_* is its weighting coefficient. GCG is an auxiliary supervision branch used only during training. It is removed during validation, inference, and model export, and therefore does not increase inference parameters or computational cost.

### 2.3. Structured Channel Pruning and Quantized Inference

Structured channel pruning used the absolute batch-normalization scale (|BN γ|) as the channel-importance score. During 50 epochs of sparsity training, an L1 gradient with a coefficient of 1 × 10^−4^ was added to candidate BN scales. P2/P3-adjacent weak layers used 0.25 times this coefficient. Layers 0, 1, 2, and 20 and the DFL layer were protected. Layers 3, 4, 16, 17, and 21–23 were pruned conservatively. Channel widths were selected from layerwise BN statistics, rounded down to multiples of eight, and constrained to retain at least eight channels. The P2-P5 detection scales, class count, regression maximum, strides, and output-point count were unchanged. Each pruned model was fine-tuned for 100 epochs using Adam. The initial learning rate was 0.001, the final learning-rate factor was 0.01, and weight decay was 0.0005. Input and augmentation settings matched the main training protocol. Because the archived main and pruned checkpoints do not provide strictly paired optimization histories, the pruning analysis characterizes structural complexity reduction rather than an independent effect on accuracy.

The 5%-pruned HCF-YOLO11n checkpoint was evaluated with PyTorch FP32 and exported to TensorRT FP16 and post-training INT8 formats. INT8 quantization required no additional model training. Weights and activations used INT8 where supported, with higher-precision fallback for unsupported operators. Calibration used the complete 832-image training split, and backend comparison used the complete 104-image test split. Export and evaluation were performed on an NVIDIA RTX 4090 using TensorRT 10.6.0.post1, PyTorch 2.1.2+cu118, and CUDA 11.8. The input size was fixed at 640 × 640 pixels with batch size 1 and a 4 GiB TensorRT workspace. Evaluation used a confidence threshold of 0.001, an NMS IoU threshold of 0.70, and a maximum of 1000 detections per image. The FP16 and INT8 engines occupied 7.6 and 7.1 MB, respectively. Network inference latency excludes preprocessing and postprocessing, whereas end-to-end latency includes both stages but excludes disk input/output and result saving.

### 2.4. Training Configuration and Evaluation Metrics

#### 2.4.1. Experimental Settings

Experiments were conducted on a server equipped with an NVIDIA GeForce RTX 4090 GPU with 24 GB of video memory (NVIDIA Corporation, Santa Clara, CA, USA) and 32 GB of system memory. The software environment comprised Python 3.10, PyTorch 2.1, and Ultralytics 8.4.146. All comparison models used the same fixed training, validation, and test partitions and an input resolution of 640 × 640 pixels. Batch-16 validation latency for the comparison and ablation experiments was divided by the number of processed images and therefore represents batched comparison throughput. Deployment-backend latency was instead measured at batch size 1, and a separate end-to-end benchmark was conducted on the Raspberry Pi 5. These latency results describe different evaluation protocols and are not directly comparable.

For the principal YOLO11n and HCF-YOLO11n comparison, both models were randomly initialized and trained from scratch for 200 epochs without pretrained weights. The common training configuration used a batch size of 16 and the Adam optimizer, with initial and final learning rates of 0.001 and 0.00001, respectively. Weight decay was 0.0005, the warm-up period was three epochs, and the early-stopping patience was 300 epochs. Automatic mixed precision and deterministic execution were enabled. The task-aligned assigner used a top-k value of 10, an alpha value of 0.5, and a beta value of 6.0. Online augmentation used a mosaic probability of 0.5, translation of 0.08, scaling of 0.20, a horizontal-flip probability of 0.5, and hue, saturation, and value gains of 0.015, 0.5, and 0.3. Mosaic augmentation was disabled during the final 20 epochs, whereas vertical flipping, mixup, cutmix, copy-paste, shear, and perspective augmentation remained disabled throughout training.

To quantify run-to-run variability, YOLO11n and HCF-YOLO11n were independently trained using three random seeds (423,407, and 2026), while the dataset partitions, optimization settings, and augmentation settings were held constant. The training subset was used for model fitting, the validation subset was used for training monitoring and checkpoint selection, and the test subset was reserved for final evaluation. For each run, the checkpoint with the highest Ultralytics validation-fitness score was retained. The three independent results were summarized using the mean and sample standard deviation.

#### 2.4.2. Evaluation Metrics

Detection evaluation used a confidence threshold of 0.001, an intersection-over-union threshold of 0.70, and a maximum of 1000 detections per image. Detection performance was evaluated using precision (*P*), recall (*R*), mAP50, and mAP50-95. The principal metrics were defined as follows:

(8) $$P=\frac{\mathrm{TP}}{\mathrm{TP}+\mathrm{FP}},$$

(9) $$R=\frac{\mathrm{TP}}{\mathrm{TP}+\mathrm{FN}},$$

Equations (8) and (9) define precision and recall, respectively. *TP* denotes correctly detected seedlings, *FP* denotes false detections, and *FN* denotes missed detections. Mean average precision (*mAP*) is defined as follows:

(10) $$\mathrm{mAP}=\frac{1}{C}\sum_{i=1}^{C}AP_{i},$$

In Equation (10), *C* denotes the number of classes and *AP_i_* denotes the average precision for class *i*. The present study considered one class, cotton seedling. The mAP50 metric represents mean average precision at an intersection-over-union threshold of 0.50, whereas mAP50-95 averages mean average precision over thresholds from 0.50 to 0.95 at intervals of 0.05 and therefore provides a stricter assessment of localization quality. AP75 was additionally reported at an intersection-over-union threshold of 0.75. Model complexity was characterized by the parameter count and floating-point operations. Inference efficiency was characterized by network inference time per image and frames per second.

Localization-specific evaluation was performed on the complete 104-image test split. Center-position error was calculated at a fixed operating point by matching predictions and ground-truth boxes one-to-one with the Hungarian algorithm. Only matched pairs with an intersection over union of at least 0.50 were retained. The Euclidean distance between the centers of each matched pair defined the center-position error, which was reported in pixels and as a percentage of the 640 × 640 image diagonal. AP75 and center-position error provide complementary measures of high-overlap accuracy and spatial displacement beyond the aggregate detection metrics.

A matched-checkpoint diagnostic was conducted to examine the localization contribution associated with GCG. The P2+LMSFF+SA-WIoU and P2+LMSFF+SA-WIoU+GCG variants were evaluated on the same complete 104-image test split. Both variants were trained for 200 epochs with seed 42, initialized from the same yolo11n.pt checkpoint, and used identical P2, LMSFF, SA-WIoU v3, input, and evaluation settings; the only configured difference was the addition of GCG. AP75 and center-position error were calculated using the protocol described above. Because this diagnostic used pretrained initialization, it was analyzed separately from the principal from-scratch comparison.

Image-level counting performance was evaluated on the complete 104-image test split using mean absolute error (*MAE*) and root mean square error (*RMSE*). Ground-truth counts were obtained from the annotated boxes. Predicted counts were obtained with a confidence threshold of 0.25, a non-maximum-suppression intersection-over-union threshold of 0.70, a maximum of 1000 detections per image, and a 640 × 640-pixel input. For *N* test images, *MAE* and *RMSE* were calculated using Equations (11) and (12), respectively.

(11) $$\mathrm{MAE}=\frac{1}{N}\sum_{i=1}^{N}\left|p_{i}-g_{i}\right|,$$

(12) $$\mathrm{RMSE}=\sqrt{\frac{1}{N}\sum_{i=1}^{N}{\left(p_{i}-g_{i}\right)}^{2}},$$

In Equations (11) and (12), *p_i_* and *g_i_* denote the predicted and ground-truth seedling counts for image *i*, respectively. Count bias was defined as the mean predicted count minus the mean annotated count; negative values therefore indicate undercounting.

Source-level generalization was assessed in a separate experiment. Model training and checkpoint selection used only COT-AD, comprising 842 training images and 98 validation images. All 100 Cotton-BNYQS images were excluded from training, augmentation, checkpoint selection, and calibration and were used exclusively as the external test set. YOLO11n and HCF-YOLO11n were randomly initialized and trained from scratch for 200 epochs using seed 42, a batch size of 16, 640 × 640 inputs, Adam, and the training configuration described above. Detection evaluation used a confidence threshold of 0.001, an NMS intersection-over-union threshold of 0.70, and a maximum of 1000 detections per image. Counting evaluation used a confidence threshold of 0.25. Precision, recall, mAP50, mAP50-95, AP75, *MAE*, *RMSE*, and count bias were calculated against the Cotton-BNYQS annotations.

### 2.5. Edge Deployment and Benchmarking on Raspberry Pi 5

Edge-deployment feasibility was evaluated by exporting YOLO11n and HCF-YOLO11n to NCNN and testing them on a Raspberry Pi 5 Model B with 8 GB RAM (Raspberry Pi Ltd., Cambridge, UK). The device used a 64-bit ARM processor and Ubuntu 26.04 LTS. CPU inference used FP32 precision through the Ultralytics 8.4.146 NCNN backend. Both models used 640 × 640 inputs, batch size 1, four NCNN threads, a confidence threshold of 0.25, an NMS IoU threshold of 0.70, and a maximum of 1000 detections per image. Ten test images were decoded into memory before timing and replayed cyclically. Each model underwent 100 warm-up iterations followed by 1000 timed iterations. This procedure was repeated three times with a 20 s cooling interval. End-to-end latency included preprocessing, NCNN inference, and NMS/postprocessing from decoded BGR images; disk input/output and result saving were excluded. Sustained throughput was derived from end-to-end latency. Process-level peak resident set size (RSS), system CPU utilization, and processor temperature were monitored during inference.

## 3. Experiments and Analysis

### 3.1. Comparison with Representative Detection Models

The selected models represent widely used one-stage detectors and a transformer-based detector. HCF-YOLO11n was compared with YOLOv5n [28], YOLOv8n [29], YOLOv10n [30], YOLO26n [31], RT-DETR-L [32], and YOLO11n [33]. YOLO architectures have been used extensively in UAV agricultural detection [34] and crop counting [35].Dataset split, input size, and core training parameters were matched across models. Network inference time was measured during batch-16 validation and divided by the number of processed images. This measure characterizes batched comparison throughput rather than batch-1 deployment latency. Table 2 summarizes the results.

HCF-YOLO11n achieved precision, recall, mAP50, and mAP50-95 values of 0.9118, 0.9130, 0.9354, and 0.4431, respectively (Table 2). These results exceeded YOLO11n by 5.47, 7.29, 5.10, and 3.74 percentage points. The parameter count and FLOPs were 14.18% and 14.63% lower, respectively. Under the batch-16 validation protocol, HCF-YOLO11n required 5.341 ms of network inference time per image.

Among the evaluated models, HCF-YOLO11n achieved the highest precision, recall, mAP50, and mAP50-95 while retaining a compact parameter and FLOP budget.

Under the matched from-scratch protocol, HCF-YOLO11n combined higher aggregate detection metrics with lower computational demand than YOLO11n. Section 3.2 examines whether this ranking was retained across three independent random seeds.

### 3.2. Multi-Seed Repeatability Analysis

YOLO11n and HCF-YOLO11n were independently trained from scratch using seeds 42,3407, and 2026. Data, optimization, augmentation, and checkpoint-selection settings were held constant. Table 3 reports each run together with the mean ± sample SD.

Across three independent runs, HCF-YOLO11n achieved mean precision, recall, mAP50, and mAP50-95 values of 0.9116, 0.9133, 0.9354, and 0.4430. The corresponding YOLO11n means were 0.8577, 0.8404, 0.8845, and 0.4056. Standard deviations did not exceed 0.0038 for YOLO11n or 0.0028 for HCF-YOLO11n. HCF-YOLO11n achieved the higher value for every reported metric under all three seeds, showing that the observed ranking was consistent across the evaluated initializations.

### 3.3. Visualization Analysis of Dense Cotton Seedling Scenes

**Figure 5:**
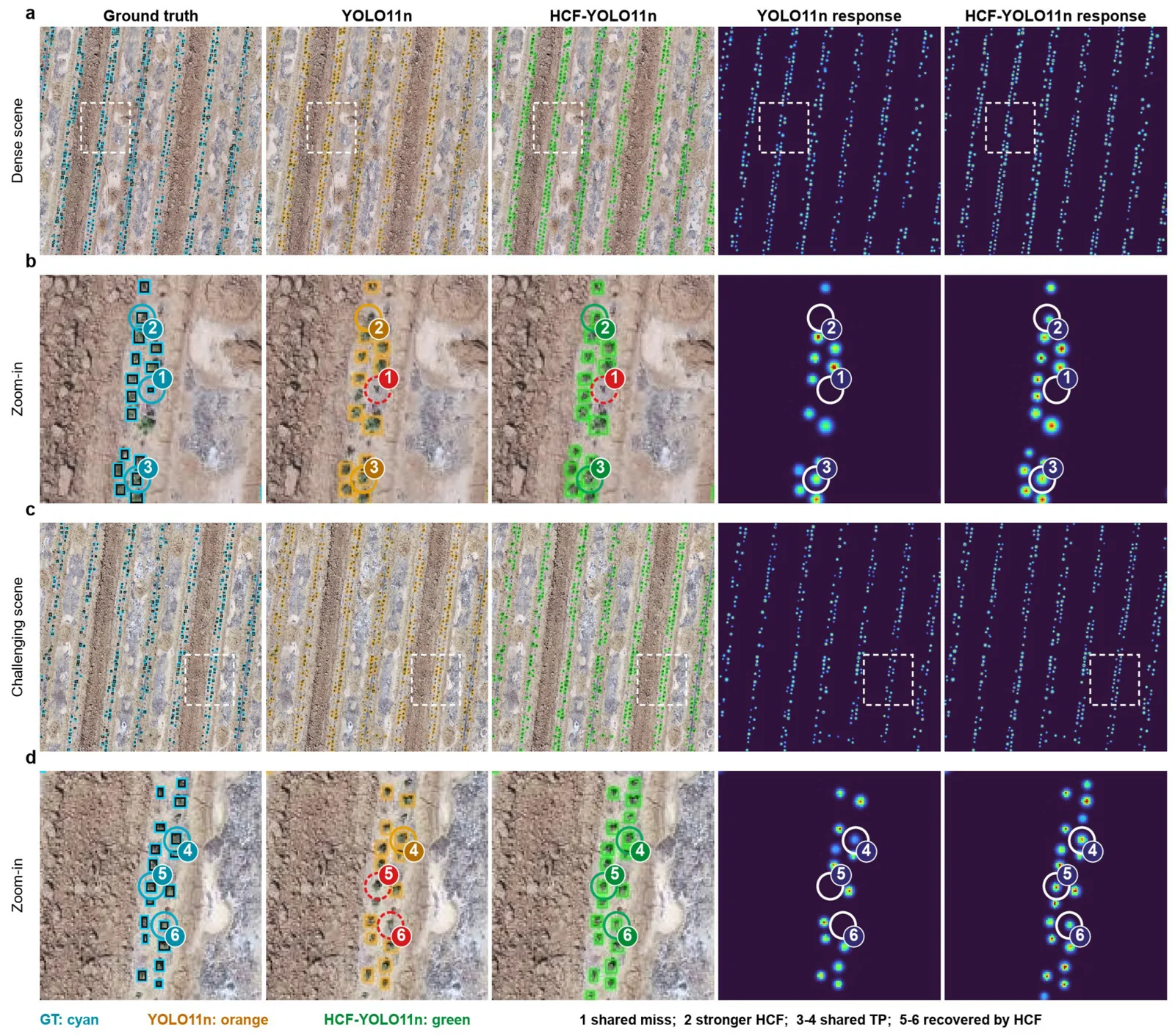
Qualitative comparison of YOLO11n and HCF-YOLO11n in dense cotton seedling scenes. (**a**,**c**) Full-scene ground-truth annotations, predictions, and detection-derived response maps for a representative dense scene and a challenging scene, respectively. Dashed white rectangles indicate the regions enlarged in (**b**,**d**). (**b**,**d**) Matched local enlargements using identical crop coordinates across all columns. Ground-truth boxes are cyan, YOLO11n predictions are orange, and HCF-YOLO11n predictions are green. Numbered circles identify success- and failure-oriented examples: 1, a seedling missed by both models; 2, a case with a stronger HCF-YOLO11n response; 3–4, seedlings detected by both models; and 5–6, YOLO11n misses recovered by HCF-YOLO11n. Response maps were generated from confidence-weighted Gaussian kernels centered on predicted boxes. The response radius was one-half of the smaller predicted-box dimension, with a minimum of three pixels, and the standard deviation was one-half of the radius, with a minimum of one pixel. Overlapping responses were combined by the pixelwise maximum. For each scene, the two response fields were jointly normalized by their shared maximum and rendered with the same color map and a display range from zero to one. No internal network activation layer was used. These panels are qualitative examples and are interpreted together with the quantitative detection metrics.

Figure 5 compares YOLO11n and HCF-YOLO11n in two traceable held-out scenes. The layout presents ground truth, identical local enlargements, and six numbered examples. These panels allow shared detections, shared misses, and detections recovered by HCF-YOLO11n to be examined in the same image regions.

On the complete 104-image test split, AP75 increased from 0.4908 for YOLO11n to 0.5139 for HCF-YOLO11n, a gain of 2.31 percentage points. With one-to-one matching at IoU ≥ 0.50, mean center-position error decreased from 0.623 pixels (SD 0.378) to 0.598 pixels (SD 0.352), a 4.06% reduction. The corresponding image-diagonal-normalized errors were 0.0688% and 0.0660%. This joint pattern indicates higher overlap accuracy and smaller center displacement on the evaluated test split. For counting, YOLO11n obtained an MAE of 33.70 seedlings per image and an RMSE of 37.99. HCF-YOLO11n reduced these values to 25.40 and 28.32, corresponding to reductions of 24.63% and 25.46%. Count bias was −33.70 seedlings per image for YOLO11n and −25.40 for HCF-YOLO11n. Both models therefore undercounted at the fixed threshold, but the undercounting magnitude was smaller for HCF-YOLO11n.

### 3.4. Ablation Study

Ablation experiments evaluated the separate and combined effects of P2, LMSFF, and GCG. All variants used the same dataset split, training settings, and test environment as the YOLO11n baseline. Table 4 reports the results.

Adding P2 increased recall and mAP50 to 0.9118 and 0.9376, respectively, but also increased parameters, FLOPs, and batch-16 network latency by 2.98%, 61.42%, and 16.41% relative to YOLO11n (see Section 3.4). Combining LMSFF with P2 reduced these three measures by 16.66%, 47.11%, and 8.68%, respectively, relative to the P2-only configuration. Thus, LMSFF substantially mitigated, but did not fully eliminate, the computational overhead introduced by the high-resolution pathway.

The GCG-only variant achieved an mAP50-95 of 0.4435. In the matched 104-image diagnostic, adding GCG increased AP75 from 0.4956 to 0.5072 and reduced mean center-position error from 0.59396 to 0.58684 pixels, corresponding to a 1.16-percentage-point AP75 gain and a 1.20% error reduction. The number of matched targets increased by 500, and mean matched IoU increased from 0.77373 to 0.78502. These consistently directed changes support an association between GCG and improved localization and detection coverage under this diagnostic setting. Because no formal hypothesis test was performed, the differences are not interpreted as statistically significant.

The complete HCF-YOLO11n model achieved precision, recall, mAP50, and mAP50-95 values of 0.9118, 0.9130, 0.9354, and 0.4431, with 2.216 M parameters and 5.388 G FLOPs. Within the single-seed ablation, P2 preserved high-resolution prediction, LMSFF reduced complexity, and GCG changed the localization and coverage metrics in a favorable direction. The matched 104-image diagnostic further associated GCG with higher AP75, lower center error, and 500 additional matched targets. These module-level results complement the complete-model comparison and do not replace it.

### 3.5. Model Compression Performance

Structured channel pruning was applied to YOLO11n and HCF-YOLO11n to assess model-complexity reduction. Table 5a reports the structural changes produced by pruning, whereas Table 5b compares PyTorch FP32, TensorRT FP16, and TensorRT INT8 implementations of the same 5%-pruned HCF-YOLO11n checkpoint under an identical evaluation protocol.

Under the same 104-image evaluation protocol, the 5%-pruned HCF-YOLO11n achieved an mAP50-95 of 0.4578, compared with 0.4431 for the unpruned reference, while reducing the parameter count from 2.216 M to 1.968 M (11.19%) and FLOPs from 5.388 G to 5.018 G (6.87%). Increasing the pruning ratio to 10% further reduced complexity but decreased mAP50-95 to 0.4290. Thus, among the evaluated pruning ratios, the 5% configuration provided the best observed balance between detection accuracy and model complexity and was selected for deployment. Its 1.47-percentage-point accuracy increase is interpreted as the combined outcome of sparsity training, structured pruning, and post-pruning fine-tuning, rather than as an effect of channel removal alone.

Table 5b compares three implementations of the same 5%-pruned checkpoint on the complete 104-image test split. PyTorch FP32 achieved an mAP50-95 of 0.4578 and required 39.769 ms for network inference. TensorRT FP16 slightly increased mAP50-95 to 0.4603 while reducing network inference latency to 1.453 ms and achieving 164.71 FPS. TensorRT INT8 achieved an mAP50-95 of 0.4509, a network latency of 1.663 ms, and 155.51 FPS. Under the tested hardware and software configuration, FP16 provided the best observed accuracy–speed balance, whereas INT8 produced the smaller engine.

### 3.6. Adaptability Across YOLO Architectures

To examine cross-architecture adaptability, the HCF components were integrated into YOLO12n and YOLO26n, producing HCF-YOLO12n and HCF-YOLO26n. Each baseline and HCF variant used the same dataset split, training configuration, and evaluation procedure within its architecture. All models were trained from random initialization. Table 6 reports detection performance and model complexity.

For YOLO12n, HCF increased precision, recall, mAP50, and mAP50-95 by 4.28, 6.45, 5.03, and 3.36 percentage points, respectively (Table 6). The parameter count and FLOPs decreased by 13.84% and 13.98%. For YOLO26n, mAP50-95 increased by 0.55 percentage points and parameters decreased by 3.41%. However, precision, recall, and mAP50 decreased by 0.59, 0.77, and 0.41 percentage points, while FLOPs increased by 24.81%. The additional high-resolution P2 computation may partly explain this cost. These results indicate architecture-dependent transfer rather than uniform gains. Further optimization is required for end-to-end detection architectures with different heads, assignment strategies, and feature-aggregation schemes.

### 3.7. Edge-Deployment Performance on Raspberry Pi 5

Table 7 reports the edge-deployment results obtained with identical NCNN settings on the Raspberry Pi 5. The values were averaged across three repeated runs.

Compared with YOLO11n, HCF-YOLO11n reduced mean NCNN inference latency from 165.45 to 142.65 ms and mean end-to-end latency from 175.36 to 149.87 ms. These changes correspond to reductions of 13.78% and 14.53%, respectively. Sustained throughput increased from 5.70 to 6.67 FPS, a 16.99% gain, while peak RSS decreased from 561.27 to 516.54 MB, a 7.97% reduction. Mean system CPU utilization decreased by 3.01 percentage points, from 86.68% to 83.67%. End-to-end latency was also less variable, with an SD of 0.91 ms for HCF-YOLO11n and 4.91 ms for YOLO11n. Under the controlled replay protocol, HCF-YOLO11n therefore showed lower latency, higher throughput, and a smaller peak memory footprint.

### 3.8. Independent External-Source Validation

After training and checkpoint selection on COT-AD only, HCF-YOLO11n retained higher performance than YOLO11n on the 100-image Cotton-BNYQS external test set (Table 8). HCF-YOLO11n achieved precision 0.8936, recall 0.8891, mAP50 0.9139, mAP50-95 0.4210, and AP75 0.4318. These values exceeded YOLO11n by 6.05, 8.26, 5.59, 3.96, and 4.57 percentage points, respectively. HCF-YOLO11n also reduced counting MAE from 41.88 to 28.74 seedlings per image and RMSE from 54.301 to 38.522, corresponding to reductions of 31.38% and 29.06%. Count bias remained negative for both models but decreased in magnitude from −16.05 to −7.96 seedlings per image.

## 4. Discussion

### 4.1. Detection Performance and Model Component Contributions

Relative to YOLO11n, HCF-YOLO11n improved detection accuracy while reducing parameters and FLOPs. This accuracy-efficiency pattern is consistent with previous lightweight YOLO, multi-scale modeling, and receptive-field-attention studies in remote-sensing imagery [36,37,38]. The three HCF components served distinct roles: P2 preserved high-resolution information, LMSFF reduced redundant computation, and GCG supplied center-region supervision. The ablation results therefore support complementary functions rather than uniform gains in every metric.

### 4.2. Qualitative Detection and Localization

The qualitative comparison includes ground truth, coordinate-matched local crops, multiple shared detections, one shared miss, two baseline misses recovered by HCF-YOLO11n, and a challenging scene. This design makes both improved coverage and remaining errors visible. Because the response maps are generated from predicted boxes rather than internal activations, they represent the spatial distribution of confidence-weighted detections and are not interpreted as evidence of learned attention.

The localization metrics provide quantitative context for Figure 5. On the complete 104-image test split, HCF-YOLO11n achieved higher AP75 and lower matched-box center error than YOLO11n. In the matched-checkpoint diagnostic, adding GCG increased AP75 from 0.4956 to 0.5072 and reduced mean center error from 0.59396 to 0.58684 pixels, an absolute reduction of 0.00712 pixels. The diagnostic also increased the number of matched targets from 37,670 to 38,170 and mean matched IoU from 0.77373 to 0.78502.

For dense images, the practical relevance of GCG is better reflected by the 500 additional matched targets and higher mean overlap than by the small per-target center-error reduction alone. The pixel-level difference does not establish a material improvement in UAV operational positioning. Moreover, this single-seed diagnostic used pretrained initialization, unlike the principal from-scratch protocol. It therefore provides supplementary checkpoint-matched evidence and should not be generalized as an independent or universal GCG effect. No formal significance test was conducted.

### 4.3. Model Compression, Deployment, and Limitations

Structured pruning reduced model complexity, and TensorRT substantially accelerated the 5%-pruned checkpoint relative to PyTorch FP32. TensorRT FP16 provided the best observed accuracy–speed balance on the RTX 4090. INT8 produced a slightly smaller engine but no additional speed advantage, showing that lower numerical precision does not guarantee higher throughput. Realized performance also depends on backend kernels, precision support, and fallback operations. The Raspberry Pi 5 experiment separately evaluated edge inference without conflating embedded end-to-end latency with the RTX 4090 backend comparison. Under controlled image replay, HCF-YOLO11n reduced end-to-end latency by 14.53%, increased throughput by 16.99%, and reduced peak RSS by 7.97%. However, images were predecoded, acquisition and storage costs were excluded, power was not measured, and no in-flight UAV test was performed. Camera capture, motion, communication overhead, power draw, and thermal behavior therefore require onboard validation.

### 4.4. Reproducibility and Scope of Evidence

YOLO11n and HCF-YOLO11n were randomly initialized and trained from scratch without pretrained weights. Repeatability was assessed through independent runs with seeds 42,3407, and 2026 while all other settings were held constant. Table 3 reports the mean ± sample SD. The three repeated Raspberry Pi measurements characterize inference-time repeatability and are distinct from the repeated training experiment.

Complete-model localization analysis reported AP75, center-position error, and image-level counting MAE and RMSE on the 104-image test split. The GCG matched-checkpoint diagnostic used the same split but only one seed and no formal significance test. Localization metrics were not recomputed for every ablation variant. The study also did not report COCO APsmall, density-stratified accuracy, inter-seedling-spacing performance, or groups defined by occlusion, illumination, and flight altitude. Missing-seedling recognition and replanting decisions were outside the scope of this study, although the detected seedling locations and counts may support such downstream analyses.

The source-isolated experiment reduced the likelihood that the model ranking was unique to the combined-data split. After training and model selection on COT-AD only, HCF-YOLO11n retained higher detection accuracy and lower counting error than YOLO11n on all 100 Cotton-BNYQS images. Nevertheless, this experiment used one seed and one external public source. Incomplete field, flight, and sequence identifiers also prevent a strict claim of generalization across independent fields or flights. Future studies should use multiple acquisition-grouped fields, repeated seeds, and prespecified operating thresholds. In addition, cultivar identities were not consistently available for the two public sources, so performance could not be stratified by cotton variety. Future evaluations should record cultivar metadata and test whether varietal morphology changes detection accuracy or localization error.

## 5. Conclusions

Cotton is a globally important fiber and cash crop, and reliable seedling monitoring supports timely field management and sustainable production [1]. This study presents HCF-YOLO for detecting small, densely distributed cotton seedlings in UAV imagery through high-resolution P2 prediction, lightweight multi-scale feature fusion, and training-only Gaussian center guidance. On the main test split, HCF-YOLO11n achieved an mAP50 of 0.9354 and an mAP50-95 of 0.4431, while reducing the parameter count and FLOPs by 14.18% and 14.63% relative to YOLO11n. The performance ranking was consistent across three independent from-scratch runs and was retained in the external-source experiment. The complete-test evaluation also showed improved high-overlap localization and lower counting error. After structured pruning, TensorRT FP16 provided the best observed accuracy–speed balance on the RTX 4090, and NCNN testing confirmed controlled edge inference on the Raspberry Pi 5. These findings indicate that HCF-YOLO balances cotton seedling detection accuracy, model size, and edge inference efficiency within the evaluated datasets and hardware protocols. The matched-checkpoint GCG diagnostic showed a consistent but small localization change and should be interpreted as supplementary evidence rather than proof of an independent operational benefit. Future work will evaluate acquisition-grouped multi-field and multi-flight datasets, quantify performance across cotton varieties, repeat external-source experiments across multiple random seeds, and measure power consumption during continuous onboard UAV operation. Further studies will investigate density-aware localization and the conversion of image-plane detections into reliable field-scale positions for practical crop monitoring.

## Figures and Tables

**Table 1:** Composition and source distribution of the cotton-seedling dataset.

Dataset Source	Image Type	Original Images	Image Proportion (%)	Annotated Instances	Instance Proportion (%)
COT-AD subset	UAV aerial images	940	90.38	63,674	62.53
Cotton-BNYQS	Cotton-field images	100	9.62	38,150	37.47
Total	-	1040	100.00	101,824	100.00

Note: The statistics represent the original images and annotations before data augmentation. Abbreviations: COT-AD, Cotton Analysis Dataset; UAV, unmanned aerial vehicle.

**Table 2:** Performance comparison among detection models.

Model	Precision	Recall	mAP50	mAP50-95	Params (M)	FLOPs (G)	Latency (ms/image, B = 16)
YOLOv5n	0.8504	0.8367	0.8816	0.4001	2.503	7.064	4.879
YOLOv8n	0.8614	0.8420	0.8877	0.4051	3.006	8.083	4.591
YOLOv10n	0.8938	0.7043	0.8054	0.3989	2.265	6.526	6.246
YOLO26n	0.8627	0.8144	0.8521	0.3503	2.495	5.187	7.296
RT-DETR-L	0.8028	0.5944	0.6774	0.3249	31.986	103.433	34.830
YOLO11n	0.8571	0.8401	0.8844	0.4057	2.582	6.311	5.446
HCF-YOLO11n	0.9118	0.9130	0.9354	0.4431	2.216	5.388	5.341

**Table 3:** Three-seed repeat-training results for YOLO11n and HCF-YOLO11n.

Model	Seed	Precision	Recall	mAP50	mAP50-95
YOLO11n	42	0.8571	0.8401	0.8844	0.4057
YOLO11n	3407	0.8615	0.8368	0.8829	0.4039
YOLO11n	2026	0.8546	0.8443	0.8861	0.4072
YOLO11n	Mean ± SD	0.8577 ± 0.0035	0.8404 ± 0.0038	0.8845 ± 0.0016	0.4056 ± 0.0017
HCF-YOLO11n	42	0.9118	0.9130	0.9354	0.4431
HCF-YOLO11n	3407	0.9089	0.9162	0.9347	0.4418
HCF-YOLO11n	2026	0.9141	0.9107	0.9362	0.4440
HCF-YOLO11n	Mean ± SD	0.9116 ± 0.0026	0.9133 ± 0.0028	0.9354 ± 0.0008	0.4430 ± 0.0011

**Table 4:** Ablation results for the proposed modules.

Model	P2	LMSFF	GCG	P	R	mAP50	mAP50-95	Params (M)	FLOPs (G)	Latency (ms/image, B = 16)
YOLO11n	×	×	×	0.8571	0.8401	0.8844	0.4057	2.582	6.311	5.446
+P2	√	×	×	0.9160	0.9118	0.9376	0.4390	2.659	10.187	6.340
+LMSFF	×	√	×	0.8670	0.8720	0.9040	0.4123	2.202	4.848	5.430
+GCG	×	×	√	0.8810	0.8780	0.9190	0.4435	2.603	6.311	5.430
+P2+LMSFF	√	√	×	0.9090	0.9070	0.9320	0.4392	2.216	5.388	5.790
+P2+GCG	√	×	√	0.9060	0.8970	0.9260	0.4350	2.664	10.187	6.340
+LMSFF+GCG	×	√	√	0.8680	0.8560	0.8970	0.4130	2.223	4.848	5.410
+P2+LMSFF+GCG	√	√	√	0.9118	0.9130	0.9354	0.4431	2.216	5.388	5.341

Note: A check mark indicates that a module was included, whereas a cross indicates that it was excluded. LMSFF denotes Lightweight Multi-Scale Feature Fusion, and GCG denotes Gaussian Center Guidance. Latency is the average per-image network inference time measured during batch-16 validation; preprocessing and postprocessing are excluded.

**Table 5:** Structural-pruning and deployment-backend evaluation for YOLO11n and HCF-YOLO11n.

(**a**) Structural and accuracy effects of channel pruning.								
**Model**	**Pruning Ratio**	**Params (M)**	**FLOPs (G)**		**mAP50-95**			
YOLO11n	0%	2.582	6.311		0.4057			
YOLO11n	5%	2.320	5.918		0.4060			
YOLO11n	10%	2.130	5.419		0.3980			
HCF-YOLO11n	0%	2.216	5.388		0.4431			
HCF-YOLO11n	5%	1.968	5.018		0.4578			
HCF-YOLO11n	10%	1.795	4.596		0.4290			
(**b**) Accuracy and inference efficiency of the same 5%-pruned HCF-YOLO11n checkpoint across deployment backends.								
**Backend**		**P**	**R**	**mAP50**	**mAP50-95**	**Inference (ms/image, B = 1)**	**Total (ms/image, B = 1)**	**FPS**
PyTorch FP32		0.9153	0.9364	0.9457	0.4578	39.769	47.044	21.26
TensorRT FP16		0.9177	0.9430	0.9510	0.4603	1.453	6.071	164.71
TensorRT INT8		0.9177	0.9397	0.4509	0.9487	1.663	6.430	155.51

Note: Panel (**a**) reports models evaluated on the same 104-image test split with identical evaluation settings. The 0% rows are unpruned reference models, whereas the 5% and 10% rows underwent sparsity training, structured pruning, and post-pruning fine-tuning. Consequently, accuracy changes relative to the 0% reference reflect the complete pruning-and-fine-tuning pipeline rather than channel removal alone. Panel (**b**) reports deployment timing for the 5%-pruned HCF-YOLO11n checkpoint with batch size 1. Inference latency excludes preprocessing and postprocessing, whereas total latency and FPS include both stages. The updated backend accuracy values were obtained on the same complete 104-image test split as Panel (**a**). Batch-1 latency is not directly comparable with the batch-16 per-image latency in Table 2 and Table 4.

**Table 6:** Performance of HCF across different YOLO architectures.

Model	Precision	Recall	mAP50	mAP50-95	Params (M)	FLOPs (G)
YOLO11n	0.8571	0.8401	0.8844	0.4057	2.582	6.311
HCF-YOLO11n	0.9118	0.9130	0.9354	0.4431	2.216	5.388
YOLO12n	0.8480	0.8293	0.8639	0.3994	2.557	6.317
HCF-YOLO12n	0.8908	0.8938	0.9142	0.4330	2.203	5.434
YOLO26n	0.8627	0.8144	0.8521	0.3503	2.495	5.187
HCF-YOLO26n	0.8568	0.8067	0.8480	0.3558	2.410	6.474

**Table 7:** Edge-deployment performance on the Raspberry Pi 5.

Model	Preprocess (ms)	Inference (ms)	Postprocess (ms)	End-to-End (ms)	P95 (ms)	FPS	Peak RSS (MB)
YOLO11n	4.58	165.45	4.94	175.36	183.27	5.70	561.27
HCF-YOLO11n	4.92	142.65	1.91	149.87	150.74	6.67	516.54

Note: Values are means across three repeated runs. End-to-end latency includes preprocessing, NCNN inference, and non-maximum suppression/postprocessing; disk input/output and result saving are excluded. RSS, resident set size.

**Table 8:** Independent external-source validation on Cotton-BNYQS after training and model selection using COT-AD only.

Model	P	R	mAP50	mAP50-95	AP75	Count MAE	Count RMSE	Count Bias
YOLO11n	0.8331	0.8065	0.8580	0.3814	0.3861	41.88	54.301	−16.05
HCF-YOLO11n	0.8936	0.8891	0.9139	0.4210	0.4318	28.74	38.522	−7.96

Note: Both models were trained from random initialization with seed 42. Detection evaluation used a confidence threshold of 0.001, an intersection-over-union threshold of 0.70, and a maximum of 1000 detections per image. Counting evaluation used a confidence threshold of 0.25 with the same intersection-over-union threshold and detection limit. Count bias represents the mean predicted count minus the mean annotated count; negative values indicate undercounting.

## Data Availability

The public datasets analyzed in this study are available from the sources cited in References [23,24], subject to their original licensing terms. The source code, model configurations, selected checkpoints, evaluation scripts, machine-readable results, dataset split manifests, preprocessing information, reconstruction instructions, and file-integrity records are available from the corresponding authors upon reasonable request. Third-party dataset images and annotations are not redistributed because their original licensing terms apply.
